# Resection of the Liver

**Published:** 1890-10-11

**Authors:** 


### RESECTION OF THE LIVER.

M. Terillon has successfully excised a portion of the liver containing hydatid cysts from a woman, aged 53, who made a rapid and complete recovery.

